# Pseudoaneurysm and arteriovenous fistula of the deep femoral artery after complete rupture of the vastus medialis muscle: endovascular treatment

**DOI:** 10.1590/1677-5449.20190001

**Published:** 2022-03-09

**Authors:** Valter Castelli, Carolina Brito Faustino, Alexandre Fioranelli, Giuliano Volpiani, Jong Hun Park, Vanessa Prado Santos, Nelson Wolosker

**Affiliations:** 1 Irmandade da Santa Casa de Misericórdia de São Paulo, Cirurgia Vascular, São Paulo, SP, Brasil.; 2 Hospital Israelita Albert Einstein, São Paulo, SP, Brasil.; 3 Universidade Federal da Bahia (UFBA), Instituto de Humanidades Artes e Ciências Professor Milton Santos (IHAC), Salvador, BA, Brasil.; 4 Universidade de São Paulo (USP), Faculdade de Medicina, São Paulo, SP, Brasil.

**Keywords:** pseudoaneurysm, A-V fistula, arteriovenous fistula, deep femoral artery, microcoil embolization

## Abstract

Due to its anatomical characteristics, the deep femoral artery is protected from most vascular injuries. We report a case of a soccer player with pseudoaneurysm of a perforating branch of the deep femoral artery, associated with an arteriovenous fistula and secondary to complete rupture of the vastus medialis muscle. Magnetic resonance imaging showed muscle damage associated with a pseudoaneurysm and angiotomography confirmed the presence of a pseudoaneurysm associated with a deep arteriovenous fistula of a branch of the deep femoral artery. Endovascular treatment of the fistula was performed by embolization with fibrous microcoils and surgical drainage of the muscle hematoma. The patient recovered well, was free from clinical complaints on the 30th postoperative day and also after 1 year.

## INTRODUCTION

The anatomic characteristics of the deep femoral artery (DFA) mean it is protected from the majority of vascular traumatisms.^1^^-^ 4 However, fractures of the femur and orthopedic surgical procedures performed to repair them can injure the DFA and its branches.^5^^,^ 6 Such injuries occur in 0.2% of surgeries and can be linked with bone fragments or iatrogenic trauma inflicted during surgical repair.^6^^-^ 8

Pseudoaneurysms of the DFA are rare and are generally associated with traumatisms. Fewer than 10% are associated with arteriovenous fistulas (AVFs).^1^

We describe a case of pseudoaneurysm of a branch of the DFA, associated with AVF, unrelated to blunt arterial trauma or iatrogeny, but caused by an uncommon mechanism of vascular injury – an injury secondary to complete rupture of the vastus medialis muscle due to extreme physical effort in a professional athlete and treated via an endovascular approach, with coil embolization.

The protocol was approved by the Ethics Committee at the Medical Sciences Faculty at the Santa Casa de São Paulo (decision number 4.976.769).

## CASE DESCRIPTION

The patient was a 35-year-old, male, professional indoor soccer player who suffered sudden, intense pain involving the anteromedial aspect of the left thigh after intense running. He was taken to an emergency service because of extreme difficulty walking. Work-up at the emergency service included magnetic resonance of the left thigh, which showed an extensive rupture of the proximal-mid third of the vastus intermedius muscle with subacute hematoma measuring 14 x 9 x 8 cm and causing local expansion effects and partial lacerations of the vastus medialis and sartorius muscles with edema. His hemoglobin level was 9.0 g/dL.

The patient was admitted, given analgesia, and put under clinical observation, with progressive easing of his pain and clinical stabilization. He remained stable and was discharged from hospital after 5 days. Three days after he had been discharged, the intense pain returned and the volume of his left thigh began to increase, to the point that walking became impossible.

He returned to the emergency service and physical examination revealed swelling and ecchymosis of the anteromedial and posterior aspects of his left thigh. Palpation of the medial aspect of the left thigh detected thrill, accompanied by an audible murmur. Left lower limb distal arterial pulses were normal.

**Figure 1:**
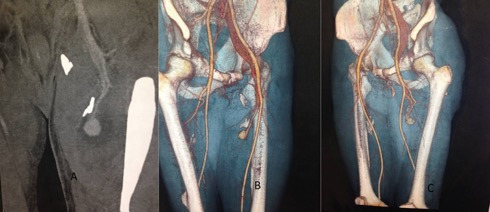
**(A)** Angiotomography of the pelvis in the coronal plane demonstrating contrasted images compatible with pseudoaneurysm of the deep femoral artery. **(B and C)** Angiotomography with 3D reconstruction showing pseudoaneurysm of a perforating branch of the left deep femoral artery associated with arteriovenous fistula.

Doppler ultrasonography showed muscle hematoma (9.2 x 4.5 x 6.5 cm) with areas of low resistance, high velocity flow from muscular vessels, compatible with an AVF in the left thigh. At this point, angiotomography was performed, revealing an AVF compromising the peripheral branch of the DFA and hematoma with an estimated volume of 567.2 cm^3^ (Figure 1).

**Figure 2:**
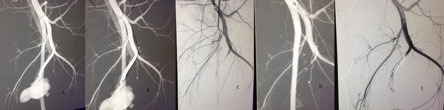
**(A and B)** Angiography showing pseudoaneurysm of a perforating branch of the left deep femoral artery. **(C and D)** Angiography with selective catheterization of the deep femoral artery showing pseudoaneurysm of a perforating branch of the left deep femoral artery associated with arteriovenous fistula, with early venous filling. **(E)** Angiography demonstrating the result of embolization of the pseudoaneurysm and the deep femoral artery branch with platinum fibered microcoils; also showing absence of early venous filling.

We decided to treat the AVF associated with the pseudoaneurysm of the branch of the left DFA using endovascular techniques. Access was obtained via a contralateral femoral puncture, followed by selective arteriography, showing the pseudoaneurysm measuring around 5 cm in a perforating branch of the left DFA, with early venous drainage, characterizing an AVF (Figure 2).

Superselective catheterization of the perforating branch was then performed with a microcatheter, followed by embolization with fibered platinum microcoils, causing occlusion of the pseudoaneurysm and exclusion of the AVF (Figure 2). Twenty-four hours after embolization, surgical drainage of the large hematoma in the left thigh was conducted. The patient recovered with no complaints during the postoperative period. He was discharged from hospital after 10 days and 30 days after discharge the hematoma and edema had regressed. After 1 year, the patient was still well and had resumed his football playing activities as normal.

## DISCUSSION

Pseudoaneurysms of the DFA are rare, which is why they are described in the literature in a few case reports, generally caused by complex fractures of the femur or during surgery to treat them.^6^^-^ 11 Blunt or penetrating traumas to the thigh region can also injure the DFA or its branches.^1^^,^ 3 ^,^ 12 In the case reported here, there had been no local blunt or penetrating trauma, but extreme physical exertion related to a complete rupture of the vastus medialis and partial rupture of two other muscles in the thigh.

Patients with pseudoaneurysms of the DFA may present with pain and edema of the thigh.^5^^,^ 8 ^,^ 11 ^,^ 12 During physical examination, a pulsating tumoral mass, thrill, and murmur can be observed in the vicinity of the fistula.^2^^,^ 3 Distal pulses should be present unless there is compartment syndrome.^1^^,^ 2 ^,^ 12 Depending on the quantity of bleeding, plasma hemoglobin levels may fall. This patient presented with a classic clinical picture.

For diagnosis by imaging, Doppler echography is considered the initial examination to use after diagnostic suspicion of pseudoaneurysm, because it is a noninvasive method.^2^^,^ 5 ^,^ 9 ^-^ 11 Angiotomography is considered the diagnostic imaging method to use after clinical suspicion.^3^^,^ 7 ^,^ 8 ^,^ 13 Angiography is the method that was most often used in the literature, because it was the imaging method most widely employed during the period when these cases were described, when noninvasive examinations were not yet routine at the majority of medial services.^1^ Our patient was initially examined with Doppler echography followed by angiotomography for diagnosis and treatment planning. Angiography was only used at the time of therapeutic intervention, which is the methodology employed currently.^5^^,^ 8 ^,^ 12

Blunt or penetrating trauma is the most common mechanism of DFA injury observed in the literature. In the present case, the patient denied having suffered any kind of trauma in the area of the thigh, only reporting intense running related to his indoor soccer profession as the factor that triggered his symptoms. In view of this, and considering the anatomic characteristics of the DFA and its branches, we related the arteriovenous involvement observed to the muscle injuries seen on magnetic resonance imaging, in particular to the ruptured vastus intermedius.

In 2015, How et al., described a case of compartment syndrome in a football player that was associated with a large hematoma of the vastus intermedius, in which, during fasciotomy, they found a partial rupture of the deep femoral vein, which they repaired surgically.^13^ Both in that case and in the case described here, there were muscle injuries, large hematoma of the vastus intermedius, and vascular injuries, although in the present case there was also arterial injury in addition to the venous damage, giving rise to the pseudoaneurysm and the AVF.^13^ The vastus intermedius is one of the muscles that make up the femoral quadriceps, the largest extensor muscle of the leg, and is fed by the lateral artery and by the medial artery emerging from the DFA.^4^ We did not find any similar reports in the literature describing a pseudoaneurysm associated with an AVF and secondary to muscle injury.

Conventional surgical repair is the treatment that was most frequently used for pseudoaneurysms secondary to arterial traumatisms reported in the literature, particularly in cases of injuries to trunk arteries.^1^ In a 2015 case of AVF associated with pseudoaneurysm, similar to ours, but caused by a stab wound, Naouli et al. chose surgical treatment with ligature of the artery and venous repair.^2^ However, for treatment of pseudoaneurysms fed by arterial branches with small diameters, embolization with endovascular techniques has been used successfully.^1^^,^ 3 ^,^ 8 Use of covered stents was also described in the literature in a case of DFA pseudoaneurysm during the postoperative period after a fractured femur.^10^ In these cases, endovascular treatment is considered a good option, since it offers good clinical results and low rates of complications.^1^^,^ 12 Our patient received endovascular treatment (embolization) followed by drainage of the large hematoma and recovered well.

Clinical follow-up is important with these patients, since there is a report of failure of coil embolization in a case of penetrating trauma with pseudoaneurysm associated with a DFA AVF, which was resolved later with surgical ligature.^3^ In our case, there was no relapse of symptoms and the patient was followed-up for 1 year with no intercurrent conditions.

In summary, occurrence of pseudoaneurysm of the DFA associated with AVF is a rare vascular traumatism diagnosis. We described a case in an athlete with an uncommon mechanism of trauma, probably the result of an injury to the vastus intermedius muscle, and which was successfully treated with microcoil embolization.
