# Supplementary material for: Pseudoaneurysm and arteriovenous fistula of the deep femoral artery after complete rupture of the vastus medialis muscle: endovascular treatment
Source: J Vasc Bras. 2022 Mar 9;21:e20190001. doi: 10.1590/1677-5449.20190001 (PMC8958435; doi:10.1590/1677-5449.20190001)
Supplement: Translation

# Translation: Pseudoaneurisma e fístula arteriovenosa da artéria femoral profunda após rotura completa do músculo vasto medial: tratamento endovascular

**Authors:** Valter Castelli, Carolina Brito Faustino, Alexandre Fioranelli, Giuliano Volpiani, Jong Hun Park, Vanessa Prado Santos, Nelson Wolosker

**Affiliations:** 1 Irmandade da Santa Casa de Misericórdia de São Paulo, Cirurgia Vascular, São Paulo, SP, Brasil.; 2 Hospital Israelita Albert Einstein, São Paulo, SP, Brasil.; 3 Universidade Federal da Bahia (UFBA), Instituto de Humanidades Artes e Ciências Professor Milton Santos (IHAC), Salvador, BA, Brasil.; 4 Universidade de São Paulo (USP), Faculdade de Medicina, São Paulo, SP, Brasil.

**Keywords:** falso aneurisma, fístula arteriovenosa, artéria femoral, embolização terapêutica

## Abstract

A artéria femoral profunda, devido às suas características anatômicas, se encontra protegida da maioria dos traumatismos vasculares. Relatamos um caso de pseudoaneurisma de ramo perfurante da artéria femoral profunda, associado à fístula arteriovenosa, secundário a rotura completa do músculo vasto medial em paciente jogador de futebol. A ressonância magnética demonstrou lesão muscular associada a pseudoaneurisma, e a angiotomografia confirmou a presença de pseudoaneurisma associado a fístula arteriovenosa de ramo da artéria femoral profunda. Foi realizado tratamento endovascular da fístula através da embolização com micromolas fibradas e drenagem cirúrgica do hematoma muscular. O paciente evoluiu bem, sem queixas clínicas no 30º dia de pós-operatório e também após 1 ano.

## INTRODUÇÃO

A artéria femoral profunda (AFP), devido às suas características anatômicas, se encontra protegida da maioria dos traumatismos vasculares^1^-^4^. No entanto, as fraturas do fêmur e os procedimentos cirúrgicos ortopédicos para suas correções podem gerar lesões da AFP e seus ramos^5^,^6^. Essas lesões ocorrem em 0,2% das cirurgias, podendo estar associadas a fragmentos ósseos ou a trauma iatrogênico durante a correção cirúrgica^6^-^8^.

Os pseudoaneurismas da AFP são infrequentes e geralmente associados a traumatismos. Sua associação com a presença de fístulas arteriovenosas (FAVs) ocorre em menos de 10% desses casos^1^.

Descrevemos um caso de pseudoaneurisma de ramo da AFP, associado a FAV, não relacionado a traumatismo contuso arterial nem a iatrogenia, mas secundário a um mecanismo incomum de lesão vascular – uma lesão secundária a uma rotura completa do músculo vasto medial decorrente de esforço físico exacerbado em atleta profissional, tratado através da técnica endovascular por embolização com molas.

O protocolo foi aprovado pelo Comitê de Ética da Faculdade de Ciências Médicas da Santa Casa de São Paulo (parecer número 4.976.769).

## DESCRIÇÃO DO CASO

O paciente, do sexo masculino, com 35 anos e jogador profissional de futebol de salão, apresentou quadro de dor súbita em face anteromedial de coxa esquerda após corrida intensa. Foi levado ao serviço de emergência devido à grande dificuldade para deambular. Para avaliação no serviço de emergência, foi realizada ressonância magnética da coxa esquerda, que evidenciou extensa rotura do terço médio-proximal do músculo vasto intermédio com hematoma subagudo medindo 14 x 9 x 8 cm, exercendo efeito expansivo regional, e lacerações parciais do vasto medial e sartório com edema. Foi detectada dosagem de hemoglobina de 9,0 g/dL.

O paciente foi internado e submetido a analgesia e observação clínica, apresentando melhora progressiva da dor e estabilização clínica. Manteve-se estável e recebeu alta hospitalar após 5 dias. Após 3 dias da alta, voltou a apresentar dor intensa e aumento de volume da coxa esquerda, que impossibilitavam a deambulação.

Voltou ao serviço de emergência e, no exame físico, apresentava aumento de volume e equimose em face anteromedial e posterior da coxa esquerda. À palpação, verificava-se frêmito, acompanhado de sopro audível, em face medial da coxa esquerda e pulsos arteriais distais do membro inferior esquerdo normais.

Foi realizada uma ultrassonografia Doppler, que demonstrou um hematoma muscular (9,2 x 4,5 x 6,5 cm), com áreas de fluxo de baixa resistência e alta velocidade dos vasos musculares, compatível com FAV em coxa esquerda. A partir desse ponto, foi realizada angiotomografia, que revelou a presença de FAV comprometendo ramo periférico da AFP, associada com hematoma com volume estimado de 567,2 cm^3^ (Figura 1).

**Figura 1:** **(A)** Angiotomografia de pelve em plano coronal demonstrando imagem contrastada compatível com pseudoaneurisma da artéria femoral profunda; **(B e C)** Angiotomografia com reconstrução 3D evidenciando pseudoaneurisma de ramo perfurante de artéria femoral profunda esquerda associado à fístula arteriovenosa.

Optamos pelo tratamento endovascular da FAV associada a pseudoaneurisma de ramo da AFP esquerda. O tratamento foi realizado através de punção femoral contralateral, seguida por arteriografia seletiva que demostrou pseudoaneurisma de cerca de 5 cm em ramo perfurante da AFP esquerda, com drenagem venosa precoce, caracterizando a FAV (Figura 2).

**Figura 2:** **(A e B)** Angiografia demonstrando pseudoaneurisma de ramo perfurante de artéria femoral profunda esquerda. **(C e D)** Angiografia com cateterização seletiva da artéria femoral profunda evidenciando pseudoaneurisma de ramo perfurante de artéria femoral profunda esquerda associado à fístula arteriovenosa, com enchimento venoso precoce; **(E)** Angiografia demonstrando resultado de embolização do pseudoaneurisma e de ramo da artéria femoral profunda com micromolas de platina fibradas, além de ausência de enchimento venoso precoce.

Foi, então, realizada a cateterização superseletiva do ramo perfurante com microcateter e embolização com micromolas de platina fibradas, gerando oclusão do pseudoaneurisma e desaparecimento da FAV (Figura 2). Passadas 24h da embolização, foi realizada a drenagem cirúrgica do volumoso hematoma em coxa esquerda. O paciente evoluiu sem queixas no pós-operatório. Recebeu alta hospitalar após 10 dias e, após 30 dias da alta, apresentava regressão do hematoma e edema. Após 1 ano, o paciente permaneceu bem e voltou a praticar atividade futebolística normalmente.

## DISCUSSÃO

Pseudoaneurismas da AFP são infrequentes, motivo pelo qual são descritos na literatura em relatos de caso esparsos, geralmente ocasionados por fraturas complexas de fêmur ou durante seu tratamento cirúrgico^6^-^11^. Traumatismos contusos ou penetrantes da região da coxa também podem gerar lesão de AFP ou seus ramos^1^,^3^,^12^. Neste caso, não houve nenhum trauma contuso ou penetrante local, mas esforço físico exacerbado relacionado à rotura completa do vasto medial e rotura parcial de outros dois músculos da coxa.

Os pacientes com pseudoaneurismas de AFP podem apresentar dor e edema de coxa^5^,^8^,^11^,^12^. Ao exame físico, observa-se a presença de massa tumoral pulsátil, frêmito e sopro na região da fístula^2^,^3^. Os pulsos distais devem estar presentes a menos que haja síndrome compartimental^1^,^2^,^12^. Dependendo da quantidade de sangramento, pode haver queda dos níveis plasmáticos de hemoglobina. Este paciente apresentou um quadro clínico clássico.

Para o diagnóstico por imagem, a ecografia Doppler é considerada como o exame inicial após a suspeição diagnóstica do pseudoaneurisma, por ser um método não invasivo^2^,^5^,^9^,^10^,^11^. A angiotomografia é considerada como o método de diagnóstico por imagem após a suspeição clínica^3^,^7^,^8^,^13^. A angiografia foi o método mais utilizado na literatura porque era o método de imagem mais utilizado na época em que os casos foram descritos, quando os exames não invasivos ainda não eram rotina na maioria dos serviços médicos^1^. Nosso paciente foi inicialmente submetido à ecografia Doppler seguida de angiotomografia, para o diagnóstico e planejamento terapêutico. A angiografia foi realizada apenas no momento da intervenção terapêutica, metodologia utilizada na atualidade^5^, ^8^,^12^.

O trauma contuso ou penetrante é o mecanismo de lesão da AFP mais observado na literatura. Neste caso, o paciente negava qualquer tipo de trauma na região da coxa, relatando apenas uma corrida intensa relacionada a prática profissional do futebol de salão como fator deflagrador dos sintomas. Sendo assim, considerando as características anatômicas da AFP e seus ramos, relacionamos o comprometimento arteriovenoso encontrado às lesões musculares evidenciadas na ressonância magnética, em especial a rotura do vasto intermédio.

How et al., em 2015, descreveram um caso de síndrome compartimental em jogador de futebol, associada a volumoso hematoma do vasto intermédio, em que encontraram, durante a fasciotomia, a rotura parcial de veia femoral profunda, corrigida cirurgicamente^13^. Tanto nesse caso quanto no caso aqui descrito, havia lesão muscular, hematoma volumoso do vasto intermédio e lesão vascular, sendo que, no caso descrito, além da lesão venosa, havia uma lesão arterial, gerando, assim, o pseudoaneurisma e a FAV^13^. O vasto intermédio é um dos músculos que compõem o quadríceps femoral, o maior músculo extensor da perna, sendo nutrido pela artéria lateral e pela artéria medial que emerge da AFP^4^. Não encontramos na literatura relato semelhante de pseudoaneurisma, associado à FAV, secundário à lesão muscular.

A correção cirúrgica convencional foi o tratamento mais frequentemente utilizado para os pseudoaneurismas secundários a traumatismos arteriais na literatura, principalmente nas lesões arteriais tronculares^1^. Em um caso de FAV associada a pseudoaneurisma, semelhante ao nosso, gerada por ferimento por arma branca, Naouli et al., em 2015, optaram pelo reparo cirúrgico através da ligadura do ramo arterial e reparo venoso^2^. Porém, no tratamento dos pseudoaneurismas nutridos por ramos arteriais de diâmetro reduzido, a embolização por técnica endovascular já foi utilizada com sucesso^1^,^3^,^8^. O uso de stents revestidos também já foi descrito na literatura em caso de pseudoaneurisma da AFP no pós-operatório de fratura de fêmur^10^. O tratamento endovascular nesses casos é considerado uma boa alternativa, visto que apresenta bons resultados clínicos e baixos índices de complicação^1^,^12^. Nosso paciente recebeu o tratamento endovascular (embolização), seguido por drenagem do grande hematoma, evoluindo adequadamente.

O seguimento clínico desses pacientes é importante, pois já houve relato de falha na embolização com molas em um caso de traumatismo penetrante com pseudoaneurisma associado à FAV da AFP, tendo-se resolvido o caso com a posterior realização de ligadura cirúrgica^3^. No nosso caso, não houve recidiva dos sintomas, e o paciente realizou o seguimento de 1 ano sem intercorrências.

Em síntese, a ocorrência de pseudoaneurisma da AFP associado a FAV é um diagnóstico raro entre os traumatismos vasculares. Descrevemos um caso com mecanismo de trauma infrequente, provavelmente resultante de uma lesão muscular do vasto intermédio em um atleta, tratado com sucesso através da embolização com micromolas.
